# Rechallenge with gefitinib following severe drug-induced hepatotoxicity in a patient with advanced non-small cell lung cancer: A case report and literature review

**DOI:** 10.3892/ol.2013.1756

**Published:** 2013-12-11

**Authors:** XUEQIN CHEN, YUELONG PAN, SHIRONG ZHANG, DADONG CHEN, SHAOYU YANG, XIN LI, SHENGLIN MA

**Affiliations:** Department of Medical Oncology, Nanjing Medical University, Affiliated Hangzhou Hospital (Hangzhou First People’s Hospital), Hangzhou, Zhejiang 310006, P.R. China

**Keywords:** hepatotoxicity, epidermal growth factor, tyrosine kinase inhibitor, adverse drug reactions

## Abstract

Gefitinib has come to be the most widely used epidermal growth factor receptor-tyrosine kinase inhibitor in the treatment of advanced non-small cell lung cancer (NSCLC) in Asian patients. Common side effects include mild to moderate skin rash and diarrhea, however, drug-induced liver injury of varying severity is overlooked in long-term gefitinib administration and rarely reported. The current case report presents a female Chinese NSCLC patient who developed severe gefitinib-induced hepatotoxicity and was rechallenged with gefitinib following a 3-month break. The patient achieved partial clinical remission but developed drug-induced grade 4 hepatotoxicity following gefitinib administration for 14 months. As an alternative, 4 cycles of chemotherapy were administered to control tumor progression. Following restoration of the patient’s liver function, gefitinib was rechallenged together with active hepatoprotective therapy. The patient presented good disease control and maintained normal liver function for >6 months. Thus, sequential chemotherapy and gefitinib rechallenge with hepatoprotective therapy may be a potential new treatment strategy for gefitinib-induced hepatotoxicity.

## Introduction

Gefitinib is a selective epidermal growth factor receptor-tyrosine kinase inhibitor (EGFR-TKI), which has come to be the most widely used in advanced non-small cell lung cancer (NSCLC) patients. It is a standard first-line therapy for advanced NSCLC patients harboring somatic EGFR mutations (1). Previous clinical studies have demonstrated that gefitinib is capable of significantly prolonging progression-free survival (PFS) and improving the quality of life in NSCLC patients carrying EGFR mutations, compared with standard chemotherapy (2–4). Due to its high binding selectivity, the toxicity of gefinitib is relatively low. The most common adverse effects are mild to moderate skin rash, diarrhea and fatigue. However, liver injury of varying severity has also been reported (5–6) and was noted in the current case report. Furthermore, the incidence of increased alanine aminotransferase (ALT) levels is more common in patients harboring somatic EGFR mutations (4,7). The development of a method to effectively utilize the clinical benefit of gefitinib, whilst simultaneously avoiding its hepatotoxicity, is a challenge for oncologists. The current case report presents a favorable clinical outcome of gefitinib rechallenge in a female Chinese NSCLC patient. Written informed consent was obtained from the patient.

## Case report

**Figure 1:**
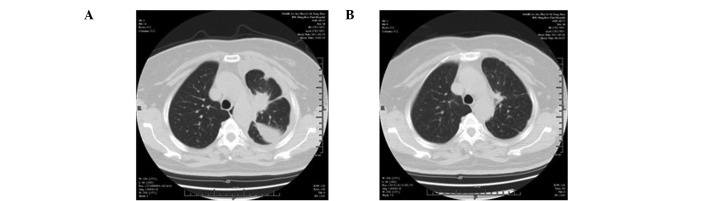
(A) Prior to gefitinib treatment, chest CT indicated a mass in the superior lobe of the left lung, encapsulated effusion in the left pleural cavity and atelectasis in the left lung. (B) Eight weeks after gefitinib treatment, CT revealed a marked decrease in lung lesion size and the disappearance of the hydrothorax. CT, computed tomography.

**Figure 2:**
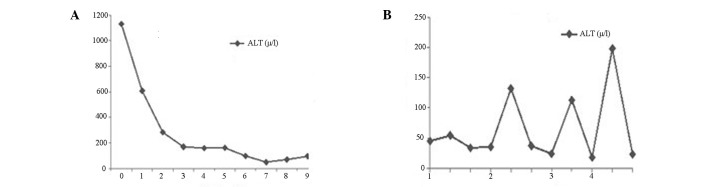
Changes in ALT during (A) hepatoprotective therapy and (B) chemotherapy. ALT, alanine aminotransferase.

A 61-year-old non-smoking Chinese female was admitted to the Affiliated Hangzhou Hospital of Nanjing Medical University (Hangzhou, China) with a repeated cough, expectoration and shortness of breath for >1 month. Chest computed tomography (CT) revealed a mass in the superior lobe of the left lung, encapsulated effusion in the left pleural cavity and left atelectasis (Fig. 1A). Cancer cells had previously been identified in the hydrothorax. A left lung biopsy was completed and histopathological study revealed an adenocarcinoma with bronchoalveolar features. Genetic analysis detected a deletion in exon 19 of the EGFR gene. The diagnosis was left lung cancer pleural metastasis (stage IV). The patient was administered gefitinib (Iressa; 250 mg per day; AstraZeneca, London, UK) as first-line therapy, following drainage of the pleural cavity effusion. Following the administration of gefitinib for 8 weeks, CT revealed an apparent decrease in the size of the lung lesion and a disappearance of the hydrothorax (Fig. 1B). Using RECIST 1.1 (EORTC, Brussels, Belgium), an evaluation of a partial response was made. The patient was prescribed long-term administration of gefitinib and followed up every 2 months with a chest CT and liver function analysis. The general condition of the patient remained good and disease was stable up to 14 months, in which severe hepatotoxicity was noted, with increased ALT and aspartate aminotransferase levels at 1,130 U/l (grade 4 toxicity) and 629 U/l (grade 3 toxicity), respectively (CTCAE v3.0; Cancer Therapy Evaluation Program, Bethesda, MD, USA). Liver metastasis and other diseases were ruled out and no other medications were administered. As such, elevation of the hepatic enzymes was attributed to gefitinib. Consequently, gefitinib treatment was discontinued and hepatoprotective therapy was carried out. Liver function later improved, with the ALT levels decreasing to 98 and 69 U/l at 6 and 10 weeks following gefitinib discontinuation, respectively (Fig. 2A). However, gefitinib withdrawal for 2 months resulted in disease progression. With regard to gefitinib-induced severe hepatotoxicity, the treatment plan was changed to chemotherapy with pemetrexed and cisplatin for four cycles, with continued hepatoprotective treatment and liver function monitoring. Lab results revealed that liver function deteriorated following each chemotherapy cycle, with ALT levels not exceeding 198 U/l, but was quickly restored to normal levels (Fig. 2B). CT examination suggested that the lesion of the left lung mildly decreased and an evaluation of disease stability was made. As the liver function recovered, gefitinib was re-administered at the same dosage, following discontinuation for 3 months. At the time of writing, the patient had been re-administered with gefitinib for 6 months. The disease remains well-controlled and hepatic enzyme levels have remained normal.

## Discussion

EGFR-TKIs inhibit TK self-phosphorylation by competing with adenosine triphosphate for the binding sites of the TK domain, thereby blocking the transmission of downstream signaling and inhibiting tumor cell proliferation (8). It has been suggested that advanced NSCLC patients harboring somatic EGFR mutations exhibit a higher response rate and longer PFS when treated with a TKI (gefitinib or erotinib), compared with chemotherapy (2,9). However, drug-induced hepatotoxicity can be a potential hindrance in the clinical administration of gefitinib. In several phase III clinical trials, it was reported that 20–30% of NSCLC patients with unknown EGFR mutation status developed hepatic enzyme elevation while receiving gefitinib treatment. Among them, grade 3–4 hepatotoxicity was observed in 3–5% of cases, with a maximum incidence of 11.33% (7,10,11). In NSCLC patients harboring identified EGFR mutations, the incidence of gefitinib-induced hepatic injury was even greater, with 50–70% of patients developing drug-induced liver toxicity and 16–26% of these with grade 3–4 injury (3,4). However, long-term administration in NSCLC is required to effectively control the disease and premature drug discontinuation will increase the risk of tumor progression.

To date, only a small number of studies on TKI-induced hepatotoxicity have been reported, which are retrospective studies with small sample sizes or case reports. A number of these suggest that gefinitib-induced liver injury may be prevented through adjusting the dosage and schedule or altering the treatment strategy, for example switching to another TKI, such as erlotinib. Seki *et al* reported a case of a 61-year-old Japanese female with lung adenocarcinoma who developed gefitinib-induced grade 2/3 liver injury following two cycles of gefitinib administration. The drug was discontinued despite complete remission of primary and metastatic tumors. However, when the patient received gefitinib at a volume of 250 mg once every 5 days, liver function was maintained at grade 1 and the disease was controlled (12). Additionally, Ku *et al* presented two patients with NSCLC who successfully switched to treatment with another TKI, erlotinib, following development of grade 2/3 gefitinib-induced hepatotoxicity (6). Furthermore, one patient demonstrated a long and near-complete response from erlotinib. In the current case report, the patient developed grade 4 liver injury during the initial gefitinib treatment. Four sequential cycles of chemotherapy were immediately administered following withdrawal of gefitinib, and the subsequent gefitinib rechallenge, combined with liver-protective therapy, was carried out following restoration of liver function. At the time of writing, gefitinib re-administration had continued for six months. The patient tolerated the initial dosage and schedule without any detectable hepatic injury, and presented good disease control with a relatively improved clinical outcome compared with the previous report (12). Sequential chemotherapy and subsequent reinitiated gefitinib treatment may be a potential new strategy for gefitinib-induced hepatotoxicity.

In conclusion, the current case report indicates that routine monitoring of liver enzymes and active hepatoprotective treatment are essential for patients receiving long-term gefitinib administration. With drug-induced liver injury, early withdrawal of gefitinib medication in NSCLC patients, without subsequent gefitinib rechallenge, is not recommended. Future studies focusing on the mechanism of TKI-induced hepatotoxicity, as well as the relationship between genetic factors and the diverse toxic effects of TKIs in the general population, are likely to promote the application of EGFR-TKIs in NSCLCs.
